# The 2022 American Diabetes Association Meeting

**DOI:** 10.1111/1753-0407.13297

**Published:** 2022-07-16

**Authors:** Zachary T. Bloomgarden

**Affiliations:** ^1^ Division of Endocrinology, Diabetes and Bone Disease, Department of Medicine Icahn School of Medicine at Mount Sinai New York USA

This summary of posters and oral reports from the 2022 American Diabetes Association Meeting, although necessarily somewhat arbitrary, offers a sense of important coming directions in the understanding and treatment of diabetes.

## 1 OBESITY APPROACHES

Doran et al. randomized 150 adults with type 2 diabetes (T2D) and HbA1c 6–6.9 to <40 g dietary carbohydrate vs usual diet, leading at 6 months to −0.23% fall in HbA1c, 10.3 mg/dl fall in fasting glucose, and 5.9 kg greater reduction in body weight,^1^ an important approach that we underutilize in clinical practice. On the other side of the spectrum of weight management, Monte et al. compared 103 patients with T2D and diabetes undergoing vertical sleeve gastrectomy and Roux‐en‐Y gastric bypass (RYGB) showing generally similar HbA1c and systolic blood pressure reduction, with somewhat greater total and low‐density lipoprotein (LDL) cholesterol reduction in the RYGB group at 6, 12, and 24 months post surgery.^2^ The importance of ectopic fat deposition in diabetes was quantitated by Eichert et al., with noncontrast magnetic resonance imaging scans of 138 persons with T2D showing liver steatosis and/or fibroinflammation in 73%, decreased aortic distensibility in 67%, kidney steatosis and/or fibroinflammation in 64%, and abnormalities in the spleen and pancreas as well, all correlating with each other.^3^

## 2 HYPOGLYCEMIA AND CONTINUOUS GLUCOSE MONITORING

Spanakis et al. randomized 18 persons with type 1 diabetes (T1D) and 155 with T2D administered insulin during general medicine and surgery hospitalization to blinded (insulin dose adjustment with point of care capillary glucose testing) versus unblinded (insulin adjustment with daily review of continuous glucose monitoring pattern, hypoglycemia alarms set at 80 mg/dl) use of Dexcom G6, finding 47% reduction in glucose <70% and 63% reduction in glucose <54 among those who experienced at least one episode of hypoglycemia, without difference in mean glucose or total insulin dosages.^4^ Soeholm et al. compared self‐reported and continuous glucose monitoring‐detected nocturnal hypoglycemia in 54 persons with T1D, finding that sensor‐detected hypoglycemia appeared to have little adverse effect, whereas episodes of which the person was aware were associated with decreased sleep quality and worse mood and alertness the following day.^5^ Brown and Aronson reported a randomized trial of 82 persons with T2D not receiving insulin, all receiving diabetes self‐management education, finding time in the 70–180 mg/dl range over 16 weeks 64% versus 76% and time above range 32% versus 22% in control patients versus those randomized to addition of Flash glucose monitoring, and HbA1c 8.1% versus 7.6%, respectively.^6^ Shah et al. examined discordant results of HbA1c and the glucose management indicator (GMI) based on 15‐day mean glucose on Dexcom G6, finding that the GMI overestimated HbA1c by approximately 0.5% in 153 nondiabetic persons and, in 93 persons with T1D, among those having HbA1c < 6.5%.^7^

Phillips et al. compared metformin‐treated patients randomized to addition of glimepiride versus insulin glargine over 4 years in the Glycemia Reduction Approaches in Diabetes (GRADE) study; during the initial 3 months hypoglycemic symptoms or documented glucose<70 mg/dl occurred in 33% versus 15%, and over 4 years severe hypoglycemia occurred in 2.3% versus 1.4%, with 50% versus 39% having HbA1c > 7.5%, suggesting basal insulin to be superior to the sulfonylurea.^8^ Andreasen et al. compared 45–60 min hyperinsulinemic hypoglycemic and euglycemic clamp studies in 24 persons with T1D, finding prolongation of the QTc interval that failed to normalize during a subsequent period of euglycemia or hyperglycemia, perhaps increasing the risk of cardiac arrhythmia or sudden cardiac death lasting beyond the time of hypoglycemia.^9^ In a promising potential approach, Laugesen et al. compared 24 adults with insulin pump‐treated T1D using continuous glucose monitoring in a randomized crossover study of 2‐week use of a dasiglucagon 80 mcg pen versus ingestion of carbohydrates for prevention and treatment of impending and actual episodes of hypoglycemia; the interquartile range of participant administration of dasiglucagon was 7–15 times during the 2‐week period, with significantly lower daily carbohydrate intake of 172 versus 192 g, and with 96% of participants indicating that they would wish to continue to use the agent.^10^

## 3 CARDIOVASCULAR DISEASE AND OTHER COMPLICATIONS

Sprenger et al. followed 1738 persons with angiographically proven coronary artery disease (CAD) or sonographically proven peripheral artery disease (PAD) over 10 years, finding major adverse cardiac events (MACE) in 27%, 38%, 48%, and 58% of persons with neither T2D nor chronic kidney disease (CKD), with just T2D, with just CKD, and with both, respectively; T2D and CKD were independently associated with 1.5‐fold and 1.9‐fold increases in risk controlling for age, sex, body mass index (BMI), hypertension, cigarette history, LDL and high‐density lipoprotein cholesterol, and HbA1c.^11^ Shi et al. studied a registry database of 11 323 angiotensin‐converting enzyme inhibitor or angiotensin receptor blocker users with new‐onset estimated glomerular filtration rate (eGFR) < 30 ml/min/1.73 m^2^, of whom 2055 (18.5%) discontinued the treatment within 6 months, finding that the latter had significant 1.26‐fold greater risk of MACE and 1.26‐fold greater risk of dialysis and/or eGFR < 15/ml/min/1.73 m over the subsequent mean 4.3 years, although there was no significant effect on mortality.^12^ The authors concluded that continued use of these agents with eGFR < 30 is appropriate, although one could equally argue that unmeasured comorbidities both caused clinicians to discontinue the agents and increased likelihood of adverse outcome. On the other end of the spectrum, Mottl et al. analyzed 564 and 644 persons with youth onset T1D and T2D at mean ages 21 and 26 with mean durations 11 and 13 years, respectively, in the Search for Diabetes in Youth (SEARCH) and Treatment Options for Type 2 Diabetes in Adolescents and Youth (TODAY) studies; respective prevalences of ophthalmologic events were 0.11% and 0.40%, of CKD none and 0.06%, of neuropathy 0.11% and 0.21%, and of macrovascular diagnoses 0.08% and 0.33% over 10–13 year of observation, with overall rates 2.5‐fold higher for microvascular and 4.0‐fold higher for macrovascular disease.^13^ Horton and Snell‐Bergeon reported that in a cohort of 597 people with T1D, each 1 SD increment in HbA1c variability was associated with a 1.6‐fold increase in cardiovascular disease (CVD) events, and a similar increment in mean HbA1c was associated with a 1.4‐fold such increase.^14^

As yet, we do not have clinically useful measures of insulin resistance. Garofolo et al. reported associations of an index based on BMI, waist, gamma‐glutamyl transferase, and triglycerides in 774 persons with T1D over 11 years; those in the highest tertile of the index had nearly 3‐fold greater mortality and >3‐fold greater likelihood of CVD events.^15^ Garofolo et al. found that, among 947 persons with T2D followed for 13 years, the highest tertile of the product of baseline fasting triglyceride*glucose was associated with nearly a doubling of CVD event rates.^16^

A recent metaanalysis showed that fenofibrate reduced the need for retinal laser treatment by 23%.^17^ Morieri et al. reported that participants in the Action to Control Cardiovascular Risk in Diabetes (ACCORD) Eye Study homozygous for the rs6008845 T/T PPARA genetic variant had greater effectiveness of fenofibrate on progression of diabetic retinopathy than C/C homozygotes or C/T heterozygotes, associated with greater fenofibrate‐induced increase in serum FGF21.^18^ This group previously found in the ACCORD‐Lipid trial that T/T homozygotes (36% of participants) experienced a 51% MACE reduction in response to fenofibrate, whereas no benefit was seen for other genotypes,^19^ offering a potential approach to personalized optimization of therapy for T2D.

Fang et al. analyzed 1451 participants in the Atherosclerosis Risk in Communities (ARIC) study with diabetes, finding that the 328 incident diabetic foot infections were associated with 1.6‐, 1.9‐, and 28.8‐fold greater subsequent likelihood of CVD, death, and nontraumatic lower‐extremity amputation, respectively.^20^ Jagannathan et al. found that among 479 T2D hospitalized for a diabetic foot ulcer, the second and third tertiles of the SD of HbA1c, adjusted for factors including the baseline HbA1c, had 1.9‐ and 2.9‐fold increase in likelihood of subsequent amputation of death over 2.4‐year follow‐up.^21^ Garoufalis reported that among 202 patients with diabetic foot ulcers, those receiving topical wound oxygen had an 88% lower likelihood of hospitalization (82% with propensity‐score matching) and 71% (73%) lower likelihood of amputation than those not receiving this treatment over 12‐months.^22^

## 4 VITAMIN D

Chatterjee Montgomery et al. analyzed the association of serum 25‐OH vitamin D (25OHD) level with development of diabetes among 2362 participants in a trial of persons with high‐risk prediabetes and overweight/obesity randomized to vitamin D3 4000 IU daily versus placebo; intratrial mean serum 25OHD ≥ 40 ng/ml was associated with significant ~50% reduced risk of T2D both in Black and in White participants, although only among those with BMI < 40 kg/m^2^.^23^ Kim et al. examined serum 25OHD and its metabolites among 114 children with islet autoimmunity and 116 controls, finding that 24,25 (OH) 2D3, an inactive catabolite, was associated with greater likelihood of islet autoimmunity and that 3‐epi‐25 (OH) D3 was protective against progression to T1D among children who seroconverted at an older age, but that neither 25OHD nor 1,25 (OH) 2D3 was associated with islet autoimmunity or with T1D development.^24^

## 5 INCRETIN‐BASED THERAPIES

Mohandas et al. reported effects of long‐acting glucagon‐like peptide‐1 receptor agonists (GLP‐1RA) in 54 persons with T1D. After a mean of 24 months the treatment was associated with significantly lower HbA1c (7.1% vs. 7.8%), fewer hospitalizations for ketoacidosis (2 vs. 11), and significantly longer time in range and shorter time in hypoglycemia, with lower 14‐day mean and SD of blood glucose and with weight reduction from 191 to 184 pounds.^25^ Arslanian et al. reported a 26‐week study of dulaglutide versus placebo administration 154 to youth with T2D, showing 0.5% and 1.0% reductions in HbA1c associated with nausea, vomiting, and diarrhea, whereas placebo was associated with a 0.5% HbA1c increase,^26^ showing reduction in HbA1c but not in body weight. Heerspink et al. compared the dual gastric inhibitory peptide/GLP‐1RA tirzepatide with insulin glargine over 85 weeks in 1995 persons with T2D and baseline HbA1c 8.5% at high cardiovascular risk in the SURPASS‐4 study, finding reduction in development of micro‐ and macroalbuminuria, although no difference in decrease in eGFR,^27^ a finding similar to that reported with GLP‐1RA.

Galindo et al. reported a study randomizing 147 T2D persons with HbA1c > 9% to insulin degludec/liraglutide combination versus basal‐bolus insulin for 26 weeks. HbA1c decreased in both groups from 10.8% to 7.7%, but significantly fewer receiving degludec/liraglutide had an episode of glucose<70 mg/dl (22% vs. 36%) and the former had significant weight loss of 3.7 kg versus weight gain of 8.4 kg, albeit with significantly greater likelihood of nausea (12% vs. 1%).^28^ McCrimmon et al.^29^ compared 814 T2D persons receiving insulin glargine/lixisenatide combination versus 814 propensity score matched T2D persons receiving basal‐bolus treatment, finding somewhat lesser HbA1c decrease from 9.2%, by 0.7% versus 0.8%, and significant weight difference at 0.1 kg weight loss versus 0.7 kg weight gain.

Benson et al.^30^ reported effects of LY3305677, an acylated peptide analog of the dual glucagon and GLP‐1RA oxyntomodulin administered once weekly, in 53 nondiabetic and 24 T2D persons, finding dose‐related 2–9 kg placebo‐adjusted weight loss and 1.1%–1.5% HbA1c reduction in the T2D group. Klein et al.^31^ administered a dual GLP‐1/glucagon receptor agonist, pemvidutide versus placebo once weekly to 34 nondiabetic overweight or obese persons, showing dose‐related reduction in weight total and LDL cholesterol, triglyceride, and blood pressure levels. Olsen et al.^32^administered a dual GLP‐1/GLP‐2 receptor agonist dapiglutide versus placebo once weekly to 40 healthy persons, showing dose‐related reduction in glucose, triglyceride, and gastric emptying. Burade et al.^33^ administered HISHS‐30, a long acting agonist of gastric inhibitory peptide, glucagon like peptide‐1, and glucagon to diabetic db/db mice, showing decrease in HbA1c, body weight, triglycerides, and glucagon levels to a greater extent than tirzepatide. Urva et al. randomized 72 T2D persons to LY3437943, a long acting agonist of gastric inhibitory peptide, GLP‐1, and glucagon, dulaglutide, or placebo for 12 weeks, showing HbAc and weight loss with expected gastrointestinal adverse effects.^34^

A number of studies explored potential oral GLP‐1RA. Pratt et al. administered an oral nonpeptide GLP‐1 receptor agonist, LY3502970, to 92 healthy persons, showing reduction in glucose and body weight and decrease in gastric emptying at 1 week but not at 4 weeks; side effects included headache, nausea, and constipation.^35^ Ono et al. administered an oral small molecule GLP‐1RA, danuglipron, to 37 Japanese persons with T2D, showing 36–42 mg/dl fasting glucose reduction from baseline of 174 mg/dl, HbA1c reduction of 1%–1.5% from baseline of 8.3%, and weight loss of 2.3–2.6 kg from baseline of 79 kg over 8 weeks, with adverse effects of nausea, vomiting, abdominal discomfort, diarrhea, and headache.^36^ Pirner et al. administered an oral small molecule GLP‐1RA, RGT‐075, to 36 T2D adults treated with metformin, showing 0.6%–1.2% reduction in HbA1c (0.4% with placebo) and 2.3–4.5 kg weight loss (1.1 kg with placebo) over up to 28 days.^37^

## 6 INSULIN

Brøsen et al. randomized 149 persons with T1D who had had ≥1 severe nocturnal hypoglycemia episode during the prior 2 years to insulin degludec versus insulin glargine U100, finding coefficient of variation of continuous glucose monitoring during the night significantly lower at 35.7% versus 39.6%, with significantly lower time below 54 mg/dl at 1.8% versus 3.1%.^38^ Bergenstal et al. reported a 12‐week open‐label trial of 343 T1D persons randomized to insulin glargine 300 U/ml and degludec 100 U/ml showing similar within‐day and between‐day glucose coefficient of variation, suggesting comparable clinical benefit of these two insulin preparations.^39^Mathiesen et al.^40^ compared 1434 women with T1D during pregnancy receiving insulin aspart versus 406 receiving other bolus insulin preparations, showing significantly lower third trimester HbA1c of 6.59% versus 6.75%, but with similar propensity‐score matched likelihood of severe hypoglycemia, abortion, preterm delivery, preeclampsia, birthweight large for gestational age, and major congenital malformations.

Bue‐Valleskey et al. compared 129 insulin‐naïve persons with T2D treated with basal insulin Fc (LY3209590), a single‐chain insulin analog fused with a human IgG Fc domain, administered once weekly versus 135 receiving insulin degludec daily for 26 weeks, finding similar improvement in HbA1c, fasting glucose, and time in the range of 70–180 mg/dl.^41^ Svehlikova et al. compared once weekly insulin icodec versus daily insulin glargine administered to 43 persons with T2D titrated to fasting glucose 80–130 mg/dL; with deliberate double and triple dosing of the usual dose, finding clinically significant hypoglycemia in 40% versus 36% of subjects for deliberate double doses of icodec versus glargine and in 53% versus 70% of subjects receiving a deliberate triple dose, with greater plasma catecholamine and cortisol responses to induced hypoglycemia with the former agent.^42^ In a study of various sites of administration, Plum‐Moerschel et al.^43^ administered insulin icodec subcutaneously in the thigh, abdomen, and upper arm to 25 persons with T2D, showing similar glucose‐lowering at all sites.

## 7 SGLT2I

Abdelgani et al. administered empagliflozin versus placebo during a norepinephrine (NE) infusion with or without pharmacologic stabilization of plasma insulin and glucagon concentrations; empagliflozin caused a 31% increase in endogenous glucose production and 67% increase in NE turnover without change in plasma NE concentration with a 34 mg/dl decrease in plasma glucose and 52% decrease in insulin concentrations, with 19% increase in plasma glucagon, 29% increase in free fatty acids (FFA), and 48% increase in β‐hydroxybutyrate (BOHB) concentrations; the changes in insulin, glucagon, FFA, and ketone concentrations were blocked when insulin and glucagon were stabilized.^44^ Solis‐Herrera et al. randomized 24 persons with T2D and heart failure with ejection fraction below 45% to BOHB infusions at two levels, increasing BOHB levels to 1.2 and 2.6 mmol/L, showing an increase in cardiac output from 40% to 44% and to 48%, respectively, with increase in stroke volume and in cardiac output as well, leading the authors to conclude that plasma ketones can provide an additional fuel for the heart, perhaps explaining some of the benefit of sodium‐glucose cotransporter‐2 inhibitors (SGLT2i) treatment on cardiac function.^45^

Pasqua et al. administered placebo and empagliflozin 2.5 and 5 mg daily in a crossover study of 24 persons with T1D whose time in the 70–180 mg/dl range was ≤70% after 14 days of closed loop insulin pump treatment, finding an increase in time in range from 59% to 72% and 70%, respectively, but with increased time below 70 mg/dl with the 5 mg but not the 2.5 mg dose. Daily 24‐h insulin requirements were 0.73 with placebo but significantly lower at 0.66 and 0.65 units/kg with empagliflozin, whereas mean daily point‐of‐care ketone levels did not rise with empagliflozin,^46^ offering a role of these agents in management of T1D.

Lamprea Montealegre et al. analyzed prescriptions for 1 319 500 adults with T2D in the US Veteran's Administration Health Care System, finding that use of SGLT2i and GLP‐1RA were 13% and 9% in patients with established atheroclerotic CVD; 14% and 11% in those with heart failure; and 11% and 10% in those with CKD, respectively, and that higher atherosclerotic CVD risk was associated with lower likelihood of administration of either agent and that higher end‐stage kidney disease risk was similarly associated with lower likelihood of administration of SGLT2i, with the authors recommending, “Intensive implementation efforts are needed to prioritize prescription of these medications to patients who would derive the largest benefit.”^47^ Htoo et al. analyzed heart failure, myocardial infarction (MI), and stroke hospitalization and mortality in 105 955 pairs of propensity score‐matched empagliflozin versus GLP‐1RA initiators and 72 498 pairs of empagliflozin versus liraglutide initiators, with baseline HbA1c 9.0%, finding 38% lower heart failure hospitalization with empagliflozin and similar MI and stroke hospitalization and mortality rates.^48^ Zhuo et al. performed a propensity score matched analysis of 74 868 older persons starting a SGLT2i versus DPP4i and of 80 475 persons starting a SGLT2i versus GLP‐1RA, finding that over 6 months there were 18% and 10% reductions in atrial fibrillation.^49^ Inzucchi et al. reported that HbA1c reduction from a baseline A1c of 8.7%, compared with changes in the placebo group in the Sotagliflozin in Patients with Diabetes and Chronic Kidney Disease (SCORED) trial of the dual SGLT1 and 2 inhibitor sotagliflozin in 10 584 persons with T2D and CKD were 0.47% with baseline eGFR >45, 0.38% with eGFR 30–<45, and 0.31% with eGFR <30.^50^
